# Analysis of Stiffness Effect on Valve Behavior of a Reciprocating Pump Operated by Piezoelectric Elements

**DOI:** 10.3390/mi11100894

**Published:** 2020-09-26

**Authors:** Jangmi Woo, Dong Kee Sohn, Han Seo Ko

**Affiliations:** School of Mechanical Engineering, Sungkyunkwan University, Suwon 16419, Korea; dnwkdal1@gmail.com (J.W.); hanseoko@skku.edu (H.S.K.)

**Keywords:** micropump, reciprocating pump, cantilever valve, valve stiffness

## Abstract

This study analyzed the characteristics of a small reciprocating pump with a cantilever valve driven by a piezo actuator. Three types of valves were fabricated to investigate the effect of the valve stiffness on the pump performance and to measure the variation in the flow rate according to the frequency. The flow rate increased with the driving frequency until a certain frequency was reached, and then it started to decrease. The rise in the pressure of the pump was found to increase as the stiffness decreased. The pump performance could be clearly distinguished according to the stiffness of the valve. The observation of the valve movements revealed that the valve opening time did not change regardless of the operating frequency, but it changed with the valve stiffness. The delay in time for the outlet valve increased significantly with an increase in the frequency. It seems that the overlap of the opening time of the inlet valve and the outlet valve plays an important role in pump performance. Therefore, it is advisable to use different designs for the inlet and outlet valves, where the shape and stiffness of the valve are adjusted.

## 1. Introduction

Hydraulic pumps are used in various applications, including for robots, precision machinery, and medical applications. There is a large variation in their specifications given their widespread use [1,2,3,4], and small pump designs have progressed to handle microscopic fluids. As small pumps can handle microfluids, this technology is applied to microfluidic applications such as chemistry, medicine, biology, and molecular analysis. Meanwhile, small pumps continue to be miniaturized by adopting smart materials as actuators [5]. In recent years, smart materials such as lead magnesium niobate (PMN), lead zirconate titanate (PZT), Terfenol-D, and galfenol have been employed as the driving power source of small pumps, and thus there have been great reductions in size and weight [6,7,8,9]. Among these smart materials, PZT is most widely used due to its stable performance [10,11].

Typical driving mechanisms of micropumps are reciprocating, rotary, and peristaltic. Rotary pumps require external motors, making it difficult to implement miniaturization and are not used much, and reciprocating pumps and peristaltic pumps are often used [5]. Although the peristaltic pump has a simple structure, it is suitable for delivering a small fixed amount of fluid, and a reciprocating pump is suitable for delivering a large flow [7,12,13]. The reciprocating pump that uses such a piezoelectric element is composed of a piston, a chamber, check valves, and other components. Here, the check valve plays an important role in giving the flow direction in the pump, and studies have been conducted on various types of check valves such as balls, membranes, cantilevers, wheels, and moving elements [5,14,15,16,17,18,19]. Van Lintel investigated the passive valve that controls the pressure and the flow rate of the pump in 1988 to increase the performance of the pump [20], and cantilever valves are widely used in small pumps due to their simple structure and easy fabrication. A cantilever valve is a thin plate valve with one fixed side and the other side open. Many researchers have investigated cantilever valves, and in most studies, the optimal design of the cantilever valves in the pump relied primarily on a numerical analysis [21,22,23,24,25]. In particular, to analyze the movement of the valve, the FSI (fluid–structure interaction) technique was used, which showed a low accuracy when the density difference between the working fluid and the valve was large [26,27,28,29]. Basically, the reason to use the numerical method to optimize the cantilever valve is that it is difficult to observe the inside of the pump.

In this study, an experimental study was conducted on the effect of valve stiffness on the performance of micropump. The basic working principle of the pump was analyzed and the pump was built to conduct experimental research. A cantilever valve was used for the check valve, and the valve stiffness was changed by adjusting the neck width of the valve, and three different stiffness designs were adopted. Basic performance measurements were made on the pump and the movement of the valve was observed using visualization techniques with a high-speed camera. The effect of the stiffness of the valve was investigated in view of the valve behavior.

## 2. Piezoelectric Pump

### 2.1. Driving Scheme

This study used piezoelectric elements that move in one direction, and it repeatedly contracts and expands depending on the applied voltage. This operation is periodically repeated to generate the flow and pressure of the pump. When the piezoelectric element expands, the fluid in the chamber is discharged to the outlet as the chamber volume contracts, and conversely, when the piezoelectric element contracts, fluid is sucked into the chamber. At this time, the fluid dynamic performance of the pump can be expressed as shown in Equation (1) [30]

(1) $$P=\frac{1}{4}Q_{max}\Delta p_{max},$$

where *P* means the total power, $Q_{max}$ is the maximum flow rate generated by the pump, and $\Delta p_{max}$ is the maximum pressure generated by the pump. This equation can be expressed as displacement and force when used as a hydraulic actuator, and pressure and flow rate when used as a pump.

At this time, the flow rate Q of the pump can be expressed as follows:

(2) Q=ηfΔV ,

where η is the efficiency of the valve, f is the dynamic frequency, and ΔV is the flow rate that occurs when the pump operates for a single cycle.

A pump operated by the frequency can be described as a vibration of the spring system. Equation (3) is a spring system that vibrates a pump piston using a piezoelectric element [31].

(3) $$\left(M_{tot}+M_{add}\right)\ddot{x}+K_{tot}\;x=\;0\;,$$

where x is the piston position, $M_{tot}$ is the mass of the moving part of the pump, $M_{add}$ is the added mass of the liquid, and $K_{tot}$ is the total stiffness of the moving part of the pump. Since the piezoelectric element moves linearly, the stiffness of the piezoelectric element is excluded from the overall stiffness. In addition, the stiffness of the valve is also excluded because the valve is operated directly by pressure. Since the fluid is incompressible for the operating range, the stiffness of the fluid is also excluded. Therefore, only the rigidity of the piston membrane is considered in the current system. In general, the stiffness is calculated by the modulus of the elasticity. However, since the force acting on the membrane is complex, the net force is used to calculate the stiffness instead of the elastic modulus. Therefore, the stiffness is expressed as follows:

(4) $$K_{m}\;=\;F_{net}⁄L_{g}\;,$$

where $F_{net}$ is the total force acting on the membrane, and $L_{g}$ is the length of the deformable region of the piston membrane. Based on this stiffness, the resonance frequency can be expressed using Equation (5).

(5) $$f_{n}=\frac{1}{2\pi }\sqrt{\frac{K_{m}}{M_{tot}+M_{add}}}\;,$$

where $M_{add}$ is the added mass, which means an additional mass due to the acceleration or deceleration of the reciprocating pump. Since it is difficult to calculate the added mass, the resonance frequency due to vibration is not easily calculated. In a pump, the resonance occurs when the driving frequency and the natural frequency coincide, and this effect affects the pump performance.

**Figure 1:**
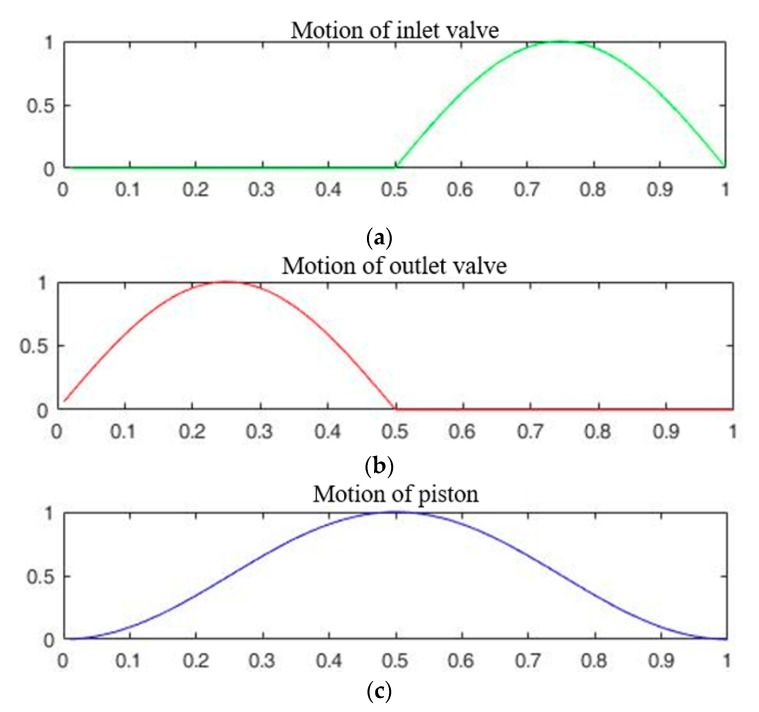
Theoretical position of the inlet and outlet valves according to the piston in the pump chamber: (**a**) inlet valve displacement; (**b**) outlet valve displacement; (**c**) piston movement.

The total amount of flow generated by the pump within a single cycle is determined by the displacement of the piston and the valve efficiency. Once the displacement is fixed, the pump efficiency is determined by the valves. The efficiency of these valves is determined by operating the valve according to the pressure generated by the pump. According to previous research, the pump has the highest efficiency when the inlet and outlet valves move immediately according to the pressure generated by the pump. Figure 1 shows the movement of the two valves. When designing the valve, the design factors are the stiffness and the shape [23]. The stiffness of the valve can be expressed by Equation (6).

(6) $$K_{v}=EA/L_{v}\;,$$

where E is the Young’s modulus of the valve, A is the area where the valve is deformed, and $L_{v}$ is the thickness of the valve. When valves of the same material are made with the same thickness, the stiffness is determined by the displacement area of the valve.

### 2.2. Pump Manufacturing

**Figure 2:**
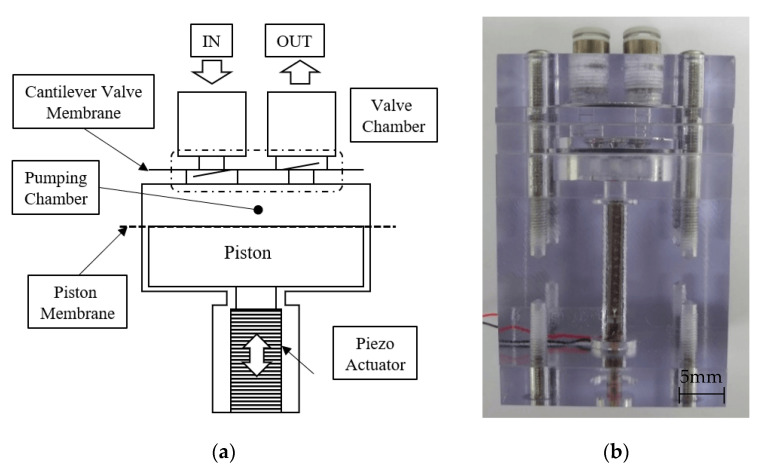
Pump design: (**a**) schematic view; (**b**) fabricated pump.

The pump used in this research is a general one-way reciprocating pump, with a structure as that in Figure 2a. The pump chamber is 30 mm in diameter and 1.5 mm in height, and one side is constructed by a membrane that makes direct contact with the piston exerted by the piezoelectric elements. The membrane not only seals the pumping chamber, but it also gives the piston a return force that the piezo elements cannot provide. In the case of this pump, the inlet and outlet valve chambers and the pumping chamber were designed to lie on a flat surface to conduct the visualization.

The pump was made of transparent PC (Polycarbonate) considering the light transmission and very similar refractive index with the working fluid. The valve and the membrane were made of PET (polyethylene terephthalate) with thicknesses of 0.2 and 0.1 mm, respectively. Figure 2b shows the appearance of the pump produced in this way. This design provides clear visibility inside the pump and easy change of the valve and piston membrane. In the process of the visualization, the shape of the part excluding the pump chamber is rectangular to minimize additional correction due to differences in the refractive index. As a piezoelectric element, model P-885.91 manufactured by PI Ceramic GmbH (Lederhose, Germany) was used, and the detailed specifications are summarized in Table 1.

**Figure 3:**
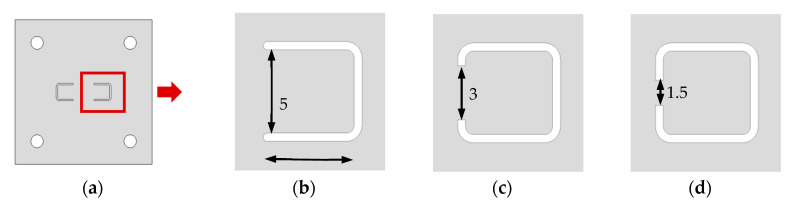
Cantilever valve (units are in mm): (**a**) top view; (**b**) Model 1; (**c**) Model 2; (**d**) Model 3.

Since the performance of the pump is greatly affected by the stiffness of the valve, three valves were designed according to the stiffness shown in Figure 3. Figure 3a shows a complete image of the valve, and Figure 3b–d shows models with various valve neck lengths. Model 1 is a cantilever type valve that is commonly used without changing the neck length. The stiffness is estimated to be 25, 16.3, and 7.5 N/µm for Models 1, 2, and 3, respectively. The stiffness is calculated from the neck thickness and length and the width of deformation which is assumed to be 0.5 mm as same as the length of the gap. Although real stiffness can be different due to the assumed deformation volume is not correct, roughly estimated stiffness can be used as a stiffness indicator.

## 3. Experimental Setup

### 3.1. Working Fluid

The fluid used in a pump is one of the most important aspects of the pump. In the previous study, the results of the analysis showed that the flow rate produced by the pump was inversely proportional to the dynamic viscosity [27,29,32,33]. In this study, a glycerin solution with a viscosity of 22.5 cP was used as the working fluid for which the concentration was 70%, corresponding to an average viscosity of the hydraulic actuator oil. The refractive index of the 70% aqueous glycerin solution was 1.43, and the refractive index of the PC was 1.5. Therefore, the error for the mismatch in the refractive index could be neglected. Detailed specifications are summarized in Table 2. 

### 3.2. Experimental Equipment

**Figure 4:**
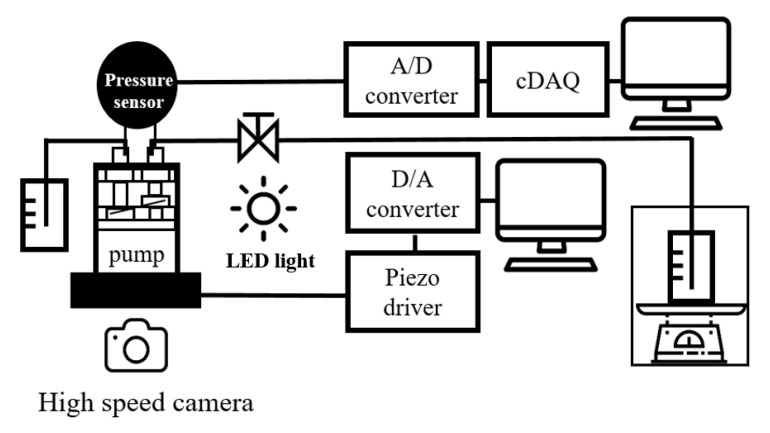
Theoretical position of the inlet and outlet valves according to the piston in the pump chamber.

The entire experimental setup was constructed as shown in Figure 4. A program was developed using LabView to operate the pump. The operating signals were created by the analog output module. (NI 9263, National Instruments, Austin, TX, USA). The operating signal from the analog output module was amplified to 100 V max using the piezo driver (E-617, PI Ceramic GmbH, Lederhose, Germany). The flow rate was measured using a balance (MW-2N (300G), CAS, Yangju, Korea), the pressure was measured using a pressure sensor (MPX5050DP, NXP Semiconductors, Eindhoven, Netherlands) with pressure response to 1 kHz, and the pressure signal was acquired using a data acquisition system (PXI-4472 module., National Instruments, Austin, TX, USA). To control the backpressure of the pump, a needle valve was placed at the outlet of the pump. To measure the current, a 10 mΩ shunt resistor was used, and the signal was acquired using the data acquisition system. For visualization, an LED light was used as the light source, and the high-speed camera (FASTCAM Mini UX50, Photron, Tokyo, Japan) was used to capture the movement of the piston and valves. The images were processed using MATLAB software. The capture rate for all frequencies was maintained at 20,000 fps. The final data was obtained via phase averaging at 32,000 frames for 1.6 s of measurement data.

**Figure 5:**
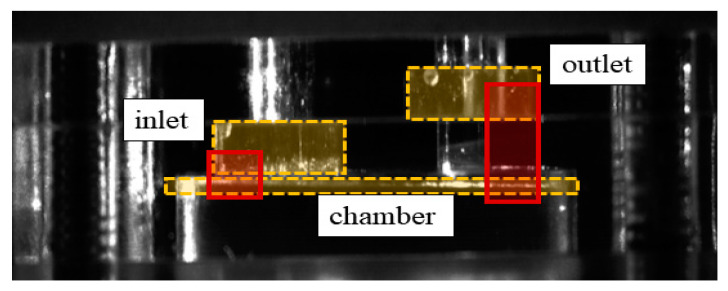
Area for visualization inside the pump.

In this study, the shadowgraphic image technique was used to analyze the movement of the valves. This technique observes the valve inside the pump by using the shadow generated by an obstruction of the light source. Although both the inlet and outlet valves were designed to be visible, both valve images could not be taken simultaneously due to the limited view of the high magnitude lens. Therefore, the motion of the two valves was taken separately including a piston and the valve movement was synchronized according to the piston movement. The actual imaging area for the valve movement is shown in Figure 5. 

## 4. Results and Discussion

### 4.1. Operating Performance

Since the performance of the pump varies depending on the driving frequency, the pressure and flow rate were measured by varying the operating frequency of the piezoelectric element. The driving frequency is 10 to 150 Hz, and the driving waveform is a sinusoidal wave.

**Figure 6:**
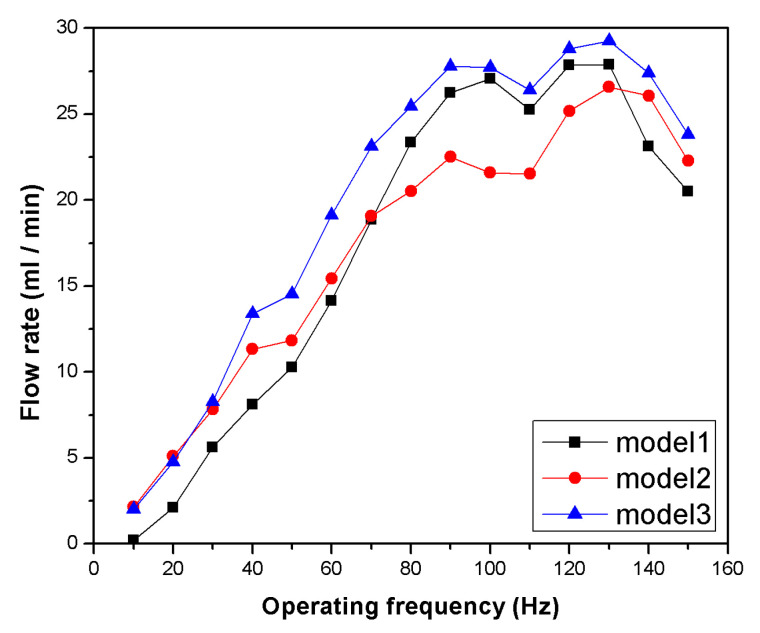
Flow rate distribution produced by a pump according to the driving frequency.

The flow rate measurement results are depicted in Figure 6. The flow rate increased as the driving frequency increased, and all valve models generated a maximum flow rate at 130 Hz. The maximum flow rate was measured to be 27.87 mL/min for Model 1, 26.57 mL/min for Model 2, and 29.25 mL/min for Model 3. In addition, the increase in flow rate changed in the 40 to 60 Hz and 80 to 120 Hz region for all three valve models. These results indicate that resonance is occurring in these regions, and the resonance is not significantly affected by the valve stiffness. Model 2 produced a lower flow rate even though the stiffness was lower than that of Model 1 for low-frequency operation. Model 3 produced a higher flow rate, with a similar trend to Model 1 except within the low-frequency region.

**Figure 7:**
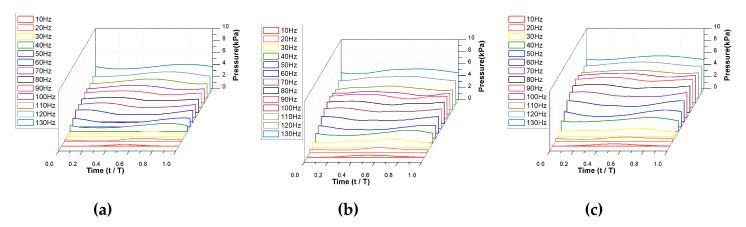
Pressure distribution generated by the pump according to the driving frequency: (**a**) Model 1; (**b**) Model 2; (**c**) Model 3.

Figure 7 depicts the pressure measurements for each valve model at a 50% backpressure. The driving signal was used as the reference time for the phase average. It can be seen that the pressure waveform shows the theoretical outlet valve opening at low operating frequencies, but changes close to a sinusoidal wave at high frequencies. The pressure signal was measured just after the outlet of the pump, so the phase delay was present. The pressure profile was not affected by the valve stiffness but the magnitude is increasing as the stiffness decreases. The maximum pressure for each valve was 4.01 kPa for Model 1, 5.12 kPa for Model 2, and 5.43 kPa for Model 3 at 130 Hz.

**Figure 8:**
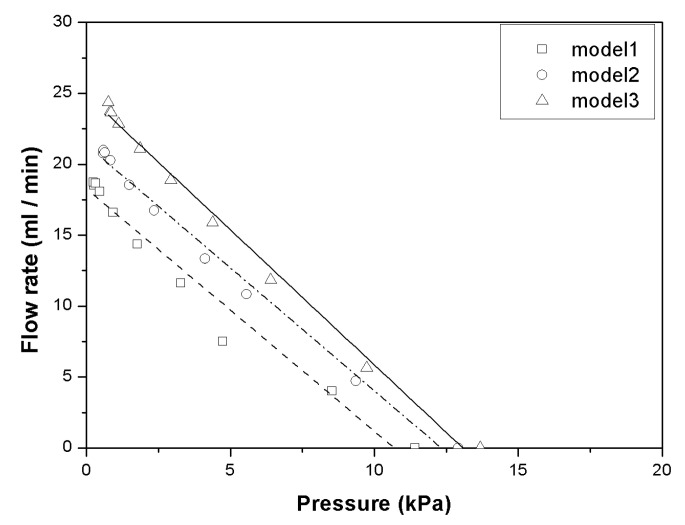
Flow rate and backpressure at 60 Hz.

The backpressure for the pump with the flow rate for each valve model was measured with a driving frequency of 60 Hz, and the results are shown in Figure 8. The maximum backpressure values were 11.41, 12.91, and 13.68 kPa for Models 1, 2, and 3, respectively. The stiffness of the valve affects the backpressure of the pump significantly, and it also affects the flow rate.

### 4.2. Valve Behavior

The previous analysis of the performance confirmed that the stiffness of the valve influenced the rise in pressure of the pump. The inside of the pump was visualized to investigate the mechanism of this phenomenon occurring inside the pump.

**Figure 9:**
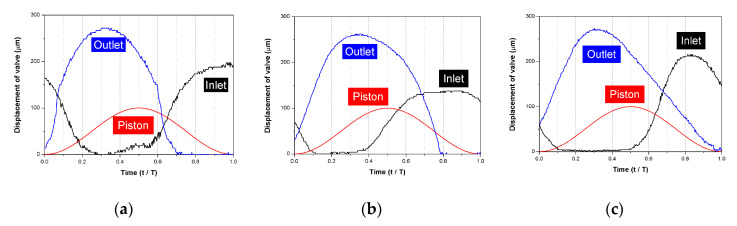
Operation of the inlet/outlet valve according to the movement of the pump piston at an operating frequency of 60 Hz: (**a**) Model 1; (**b**) Model 2; (**c**) Model 3.

Figure 9 shows the movement of the inlet/outlet valve according to the pump operation at 60 Hz. In the case of Model 1, operation follows the theoretical movement quite well except for the overwrap where the piston movement changes. It can be clearly seen that the lower the rigidity, the longer the opening time of the outlet valve and the shorter the opening time of the inlet valve. The closing time for the outlet valve is very short for Model 3. However, the overlap time does not change significantly.

**Figure 10:**
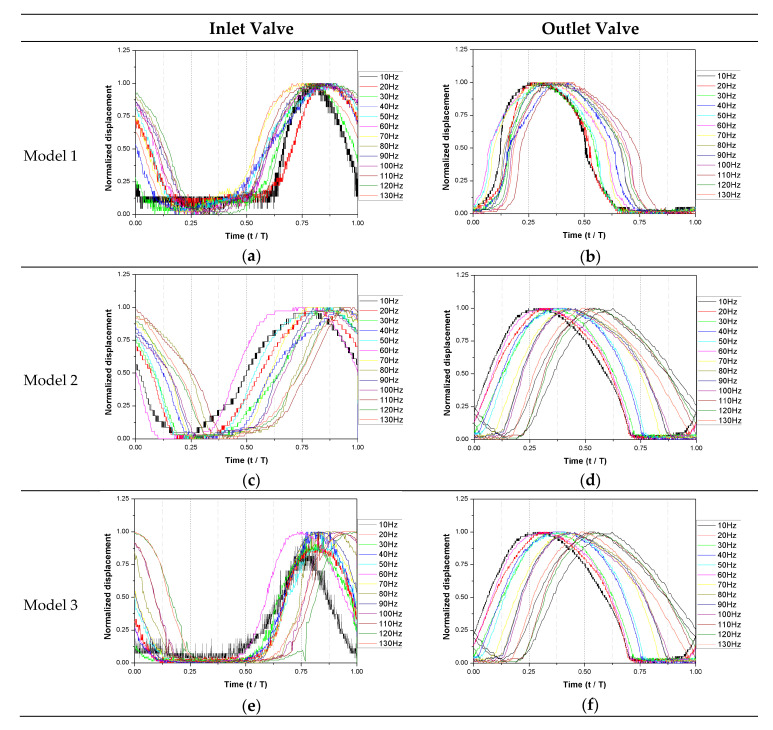
Normalized valve movement according to driving frequency. (**a**) Inlet valve-Model 1; (**b**) Outlet valve-Model 1; (**c**) Inlet valve-Model 2; (**d**) Outlet valve-Model 2; (**e**) Inlet valve-Model 3; (**f**) Outlet valve-Model 2.

The normalized valve movement according to the frequency is presented in Figure 10. The displacement is normalized by the maximum displacement of the valve and the time is normalized by the operating cycle period. In Models 1 to 3, the movement of the valve was measured by changing the frequency at regular intervals. When the frequency changed, the moving phase of the valve moved, but the movement itself maintained a constant pattern. Only a phase delay could be observed for each frequency. This result agrees well with the pressure profile measurement results shown in Figure 7. This result agrees well with the pressure profile measurement results shown in Figure 7. The opening time of the valve did not appear to be significantly related to the driving frequency, and it was related to the stiffness of the valve. 

**Figure 11:**
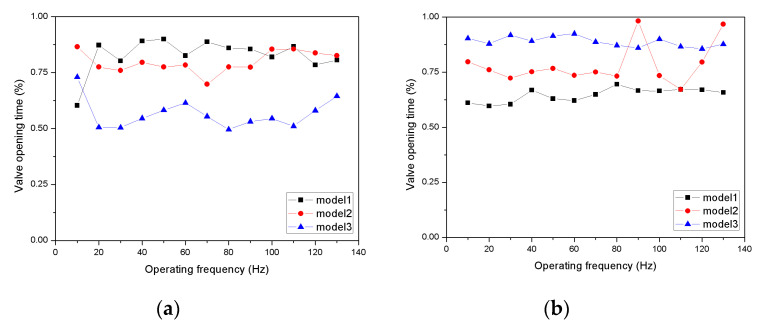
Normalized valve opening time according to the driving frequency: (**a**) inlet valve; (**b**) outlet valve.

Figure 11 shows the normalized valve opening time according to the driving frequency. The valve open time was derived from the valve open and close time which is the moment when the normalized displacement crosses 0.05 and the open time is normalized by the cycle period. When the piston was operated in a cycle, the time at which the inlet and outlet valves opened was measured. The intake valve for Model 3, which had the lowest stiffness, was measured to have a short opening time on average. The lower the stiffness, the shorter the total opening time. Conversely, for the outlet valve of Model 1, which had the highest stiffness, a short opening time was measured on average. The valve with the lowest stiffness provided the longest opening time. Reduced stiffness provided increased pressure and flow by enforcing the inlet valve to open shorter and the outlet valve longer. On the other hand, a significant pattern of the open time according to the frequency change could not be observed. The result indicates that the valve opening time is closely related to the pump performance.

**Figure 12:**
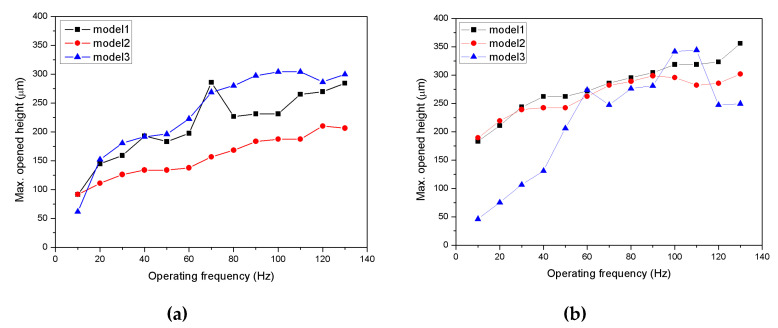
Height for the valve opening according to the driving frequency: (**a**) inlet valve; (**b**) outlet valve.

Figure 12 shows the magnitude of the inlet and outlet valve openings according to the frequency. The valve opening height tends to increase as the driving frequency increases. However, there is no distinct relationship between the stiffness and the valve opening. The inlet valve movement follows the movement of the piston well, but the outlet valve seems to decrease in height as the valve opens with a decreasing stiffness of the valve, as shown in Figure 10b. In particular, the outlet valve for Model 3 shows an unstable behavior. The reduced valve stiffness is regarded to provide an insufficient return force. A lower stiffness can be seen to produce a more opening, so the flow rate of the pump is expected to improve, but not for the outlet valve. Rather, the inlet valve is affecting the pump’s performance. Figure 10a confirmed that the inlet valve behaved the same as the flow rate generated by the pump. This suggests that it is important to increase the pressure in the chamber when driving the pump.

**Figure 13:**
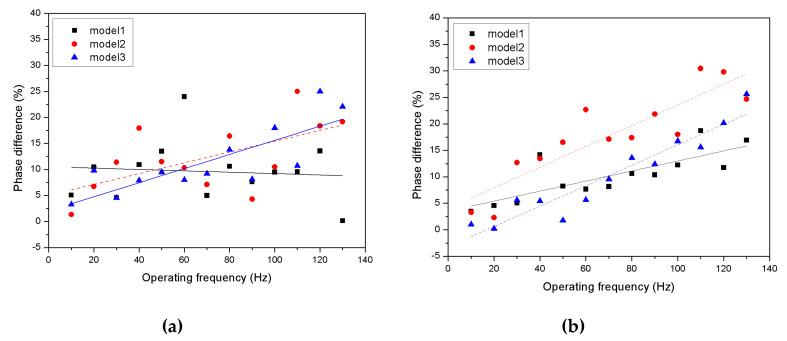
Phase difference of normalization of the inlet/outlet valve according to driving frequency: (**a**) inlet valve; (**b**) outlet valve.

Figure 13 shows the phase delay of the valve according to the driving frequency. For the inlet valve, the effect of the operating frequency on the phase delay is not clear. However, for the outlet valve, there is a somewhat proportional result. The effect of the stiffness cannot be clearly determined. The standard deviations according to the inlet valve were 5.78% for Model 1, 6.74% for Model 2, and 6.50% for Model 3. For the outlet valve, the standard deviations were 4.63% for Model 1, 8.61% for Model 2, and 7.89% for Model 3. Comparing the flow rate measurements, Model 2 showed the largest dispersion for the flow rate as well as the phase delay. This means that the movement of Model 2, which has the largest dispersion, is the most unstable to operate the pump. Although it can be confirmed from the flow rate measurements, this means that the movement for Model 2, which has the largest dispersion, is the most unstable by operating the valve. The relationship between the valve’s performance and the stiffness is not expected to be linear. The small number for Model 2 in the frequency range, where the generated flow rate is high, is presumed to be caused by the instability of the dispersion.

The stiffness is important to improve the pump performance, and the behavior of the inlet and outlet valves is different, so it is recommended to produce the corresponding design. Both valves can be designed with a reduced stiffness to allow for operation, but at the same time, an improper stiffness can induce unstable operation. Therefore, when designing a valve, both the stiffness and the valve operation must be considered.

## 5. Conclusions

This study investigated the behavior of the valves with various stiffnesses and frequencies to assess the performance in a reciprocating pump driven by a piezoelectric element. The flow rate and pressure that were measured to increase as the driving frequency increased, as expected. In addition, the backpressure of the pump was found to increase as the stiffness of the valve decreased. The effect of the resonance in the specific frequency region could be observed. The behavior of the valve according to the driving frequency of the pump and valve stiffness was analyzed using visualization techniques, and the valve opening time did not change regardless of the operating frequency, but it changed with the valve stiffness. The delay in time for the outlet valve increased significantly with an increase in the frequency. As a concluding remark, the inlet and outlet valves could have different designs to improve performance. Improvements in performance and operational stability can be expected through optimization studies on inlet and outlet valves with different stiffness.

## Figures and Tables

**Table 1:** Characteristics of lead zirconate titanate (PZT).

Model	p-885.91
Cross-sectional area (mm^2^)	5 × 5
Length (mm)	36
Capacitance (nF)	3.1
Blocking force (N)	950
Max. Displacement (µm)	38
Stiffness (N/µm)	25

**Table 2:** Properties of the working fluid.

Property	Value
Working fluid	70% aqueous glycerin solution
Density (kg/m^3^)	1181
Viscosity (cP)	22.5
